# Safety and outcomes of bikini-incision DAA for hip arthroplasty with large acetabular cups (≥56 mm): A single-surgeon series of 215 cases

**DOI:** 10.1051/sicotj/2025021

**Published:** 2025-04-14

**Authors:** Fadhil Mat Salleh, Ikram Nizam

**Affiliations:** 1 AOA Accredited Fellow, Mulgrave Private Hospital 48 Blanton Dr Mulgrave VIC 3170 Australia; 2 Ozorthopaedics, Centre for Adult Joint Arthroplasty 1356 High Street Malvern VIC 3144 Australia

**Keywords:** Direct anterior approach, Bikini incision, Total hip arthroplasty, Acetabular cup, Harris Hip Score

## Abstract

*Introduction*: This study evaluates complications associated with the bikini-incision direct anterior approach (DAA) total hip arthroplasty (THA) performed by a single surgeon on a standard operating table, with a focus on cases requiring large acetabular cups (≥56 mm). Secondary objectives include assessing clinical outcomes and implant survivorship. *Methods*: A retrospective analysis was conducted on primary bikini-incision DAA THAs performed by a single surgeon between 2013 and 2024. Cases involving acetabular cups ≥56 mm were included, while emergency hip fracture cases and those requiring posterolateral approaches were excluded. Clinical data, radiographs, and Kaplan-Meier survival analysis were used to assess complications, Harris Hip Scores (HHS), and implant survivorship. *Results*: This study included 215 THA procedures performed on 210 male patients (mean age 67 years, BMI 28.6), with an average follow-up of 3.9 years. The primary indication was osteoarthritis (88.4%). The mean preoperative HHS was 41.8, which significantly improved to 92.6 postoperatively (*p* < 0.001). Complications included lateral femoral cutaneous nerve (LFCN) neuropraxia (2.3%), periprosthetic fractures (0.93%), and femoral stem subsidence (0.93%). The revision rate was 0.93%, with Kaplan-Meier analysis indicating a 99% survival rate for the stem and 100% survival for the acetabular cup at the final follow-up. *Discussion*: The bikini-incision DAA THA using a standard operating table provides excellent short- to mid-term functional outcomes and implant survivorship for patients requiring large acetabular cups (≥56 mm). The approach is associated with low complication and revision rates, supporting its safety and efficacy in this cohort.

## Introduction

Total hip arthroplasty (THA) using the direct anterior approach (DAA) has gained popularity due to its potential benefits, including reduced postoperative pain, faster recovery, and improved functional outcomes compared to other approaches [1, 2]. The bikini-incision modification of DAA offers similar functional outcomes to the traditional longitudinal incision but with fewer wound complications and enhanced scar aesthetics, improving patient satisfaction [3].

Despite these advantages, some studies report higher rates of wound complications in obese patients, increased intraoperative fractures in osteoporotic individuals, and a learning curve that may contribute to early complications [4]. However, when performed by experienced surgeons, DAA can be executed safely without increased risk to patients [5].

The use of large acetabular cups (≥56 mm) in THA is often necessary to optimize prosthetic stability, particularly in revision cases or in patients with larger anatomical requirements [6]. However, concerns exist regarding the long-term wear and mechanical complications associated with larger cups. While studies have explored acetabular design and implant survivorship [7], limited research specifically evaluates the safety and outcomes of bikini-incision DAA for THA involving large acetabular components.

This study aims to assess the safety, complications, and functional outcomes of bikini-incision DAA THA using large acetabular cups (≥56 mm). We hypothesize that this approach is associated with low complication and revision rates, while achieving significant improvements in functional outcomes as measured by the Harris Hip Score (HHS).

This study reports the complications encountered during bikini-incision DAA THA performed by a single surgeon using a standard operating table. Additionally, it evaluates clinical outcomes and implant survivorship to determine the feasibility of this approach in patients requiring large acetabular cups.

## Material and methods

### Study design and methodology

**Figure 1:**
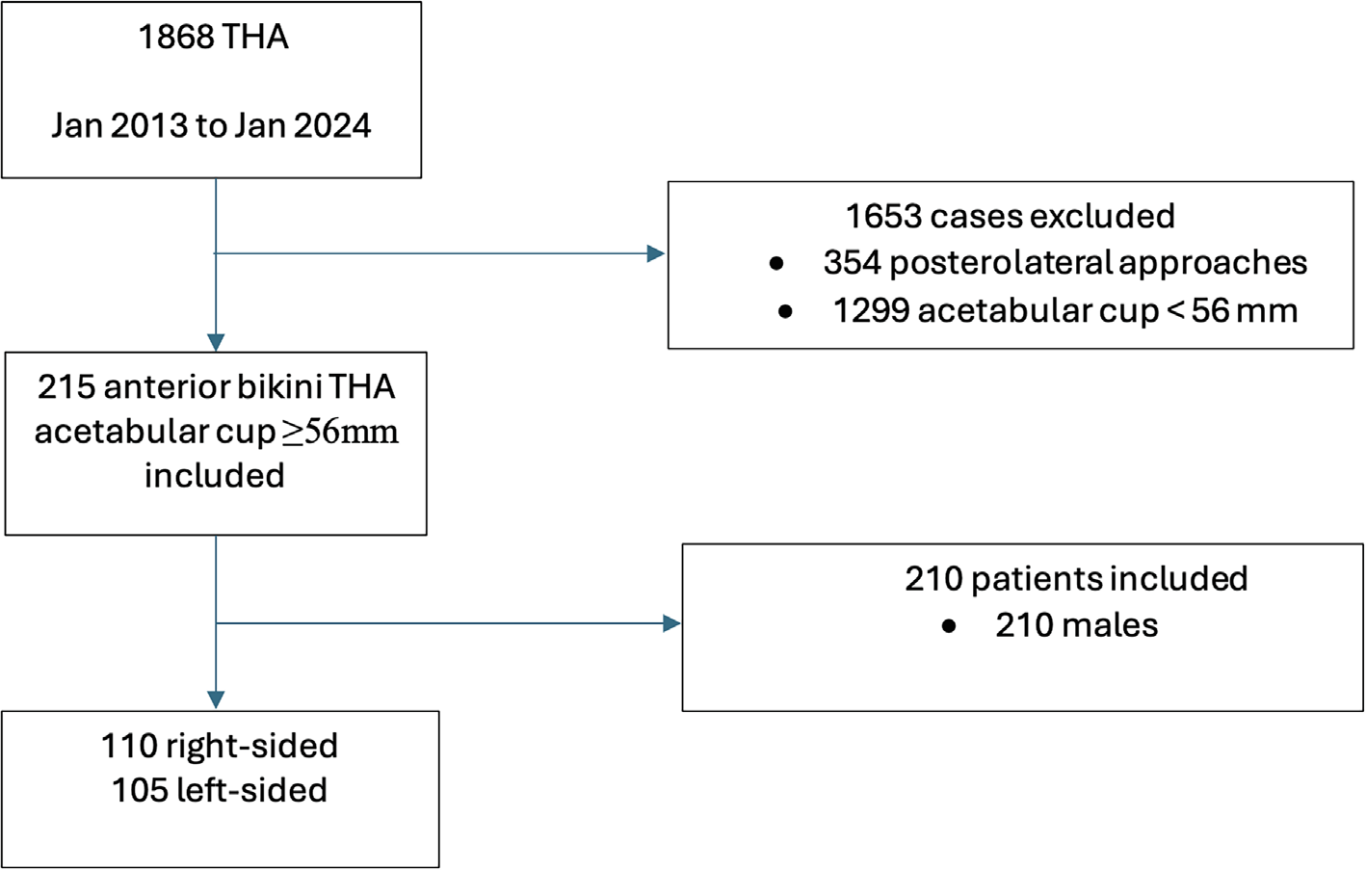
Flowchart.

This retrospective study analysed primary THAs performed by a single surgeon using a standard operating table from January 2013 to January 2024. The inclusion criteria were elective THAs utilizing the bikini incision DAA, regardless of the indication for surgery with acetabular cup sizes ≥56 mm. Cases involving posterolateral approaches for femoral neck fractures were excluded (Figure 1).

Institutional review board approval was not required for this retrospective study. Data was recorded prospectively using Genie Software Version 9, including demographics, complications, and functional outcome measures, specifically the Harris Hip Score (HHS). HHS, which combines patient-reported outcomes (PROMs) and clinician-reported outcomes (CROMs), was assessed preoperatively and during follow-ups.

Intraoperative complications, such as iatrogenic fractures, were documented through operative notes and post operative X-rays. Postoperative complications like LFCN neuropraxia and iliopsoas tendinitis were recorded during follow-ups and annual X-rays. Imaging was used to detect issues such as stem subsidence, periprosthetic fractures, joint dislocation, or implant loosening.

Radiographic measurements assessed stem subsidence and leg length discrepancies. Statistical analysis, including Kaplan-Meier survival analysis and repeated measures ANOVA, evaluated implant survivorship and differences in HHS across variables like age and prosthesis type. A literature review was conducted to contextualize complication rates in comparison to published studies.

### Surgical procedure overview

**Figure 2:**
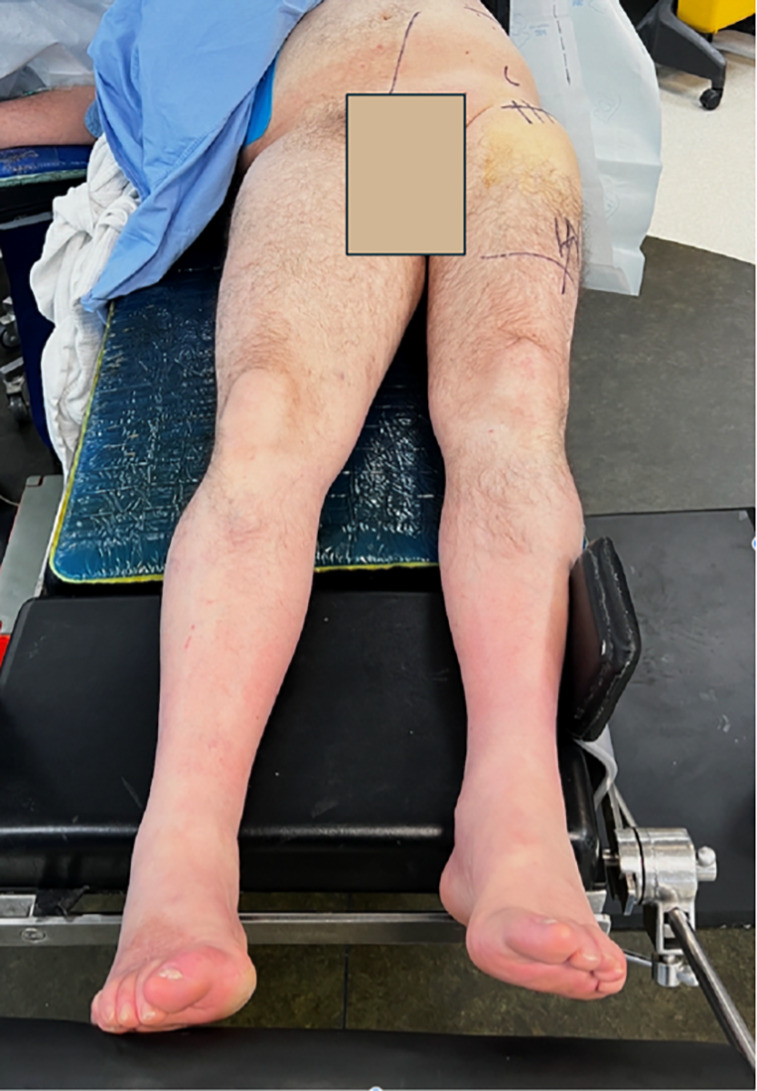
Patient positioning on a standard operating table during DAA THA, with the operating table adjusted to facilitate hip extension and proximal femur access for broaching.

All surgeries were performed by a fellowship-trained surgeon specializing in the DAA for hip arthroplasty, as previously described [4, 8]. Standard DAA patient positioning was utilized, with a regular operating table modified to allow hip extension without the need for a dedicated / fracture table or intraoperative imaging (Figure 2) [4].

The modified vessel-sparing bikini incision technique involved a 6–10 cm horizontal incision along the lateral groin crease, preserving vital structures such as the lateral circumflex femoral vessels and the anteromedial capsule [8]. The femoral head and neck were resected following capsular release, with the leg positioned in a figure-four posture to stretch the posterosuperior capsule.

**Figure 3:**
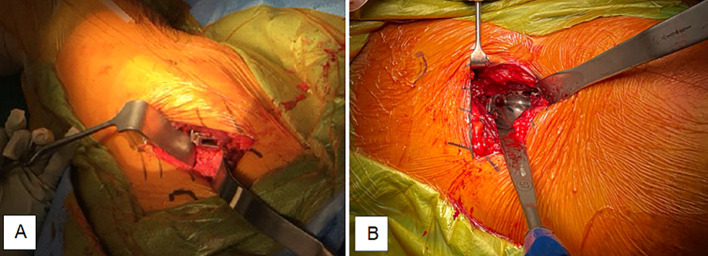
Surgeon’s view during DAA THA. (A) Proximal femur with femoral broach in situ. (B) Acetabular exposure with cup prosthesis in situ.

Utilizing the Woodpecker pneumatic broaching system, femoral preparation was conducted in a lazy figure-four position under the contralateral leg, with the operating table broken to 30–45° and the broach left in situ [4]. The acetabulum was prepared with the table levelled, and screws were typically inserted through the acetabular shell to enhance stability (Figure 3). Trial reductions assessed joint stability, and leg lengths were measured using the medial malleoli for alignment.

### Prosthetic components

Oxinium femoral heads (Smith and Nephew, Memphis, TN) were used in all cases, with head size (36 mm or 40 mm) determined by acetabular cup size (56 mm or greater). All cases utilized a highly cross-linked R3 polyethylene liner. Femoral stems included cementless Polar stems (Smith and Nephew AG, Baar, Switzerland) and CPCS cemented stems (Smith and Nephew, Memphis, TN). Cemented stems preferred for patients over 75 years or with poor bone quality. Cementation employed fourth-generation techniques using Simplex HV with Gentamycin cement (Stryker Howmedica Osteonics, USA) [4].

### Perioperative and postoperative care

Intravenous cefuroxime was routinely administered on induction and after surgery. Postoperatively, local infiltrative anaesthesia was provided through an intraarticular catheter for 24 h as per the Kerr-Kohan method [9]. Patients followed a postoperative rehabilitation protocol, which included early mobilization and discharge.

Postoperatively, patients were advised against combining hip extension and external rotation [4]. They were encouraged to mobilize within three to four hours of the surgery once spinal anaesthesia had worn off [9]. Mechanical thromboprophylaxis, including above-knee thromboembolic deterrent stockings and pneumatic calf compressors, was used in combination with chemical prophylaxis in all patients; oral Aspirin or subcutaneous Enoxaparin for six weeks [8].

### Statistical analysis

Statistical analyses were conducted using SPSS version 30 (IBM Corp., Armonk, NY). Normality of data distribution was assessed using the Shapiro-Wilk test, and homogeneity of variances was evaluated using Levene’s test. Repeated measures ANOVA was performed to compare preoperative and postoperative Harris Hip Scores (HHS), with post hoc Bonferroni corrections applied where applicable. A *p*-value of <0.05 was considered statistically significant.

## Results

### Study population and demographics

A total of 215 THAs were included in our study, performed on 210 patients, including five patients undergoing bilateral hip arthroplasties. The average follow-up period was 3.9 years (1–10.5 years). All patients were male, with an average age of 67 years (27–92 years) and an average BMI of 28.6 (21.7–44.2). Of the 215 procedures, 110 were performed on the right hip and 105 on the left. The primary surgical indication was osteoarthritis (*n* = 190, 88.4%), followed by dysplasia (*n* = 20, 9.3%), avascular necrosis (*n* = 3, 1.4%), and inflammatory conditions (*n* = 2, 0.9%).

There were 205 cementless THAs – Polar stem (95.3%) and 10 cemented CPCS stems (4.7%). The average acetabular cup size was 57.1 mm (56–62 mm), and the average femoral stem size was 4.9 (1–9). The average length of hospital stay was 1.5 days (1–3) (Table 1).

**Table 1:** Patient demographic.

	*n*	Minimum	Maximum	Mean	Std. Deviation
Age (year)	215	27	92	67.0	11.0
BMI	215	21.7	44.2	28.6	4.09
Cup size (mm)	215	56	62	57.1	1.49
Femur size	215	1	9	4.9	1.76
HHS pre	215	21	67	41.8	10.1
HHS post	215	79	100	92.6	3.9
Follow-up (year)	215	1	10.5	3.89	2.4
LOS (day)	215	1	3	1.47	0.56

BMI, body mass index; HHS, Harris Hip Score; LOS, length of stay.

### Functional outcomes

Preoperative HHS averaged 41.8 (21–67), while postoperative HHS averaged 92.6 (79–100). A significant difference was found between preoperative and postoperative HHS scores (*p* < 0.001), when analysed using repeated measure ANOVA using within-subjects effects. However, factors such as age, BMI, cup size, femoral stem size, surgical indication, and prosthesis type did not have a significant effect on the difference in HHS (Table 2).

**Table 2:** Repeated measures ANOVA.

Source	SS	df	MS	F	*p*-value
HHS	18532	1	18532	1524	<.001
Age	1.5	1	1.5	0.04	0.84
BMI	26.4	1	26.4	0.69	0.41
Surgical indication	67.8	3	22.6	0.59	0.62
Cup size	54.7	1	54.7	1.43	0.23
Stem size	14.6	1	14.6	0.38	0.54
Prosthesis	10.1	1	10.1	0.26	0.61
Error	7839	205	38.2		

**Legend**: The within-subjects effects test reveals a significant difference between preoperative and postoperative Harris Hip Scores (HHS). However, no significant differences were found in the between-subjects effects for age, BMI, cup size, femoral stem size, surgical indication, or prosthesis type regarding changes in HHS.

### Complications and revision rate

Intraoperative complications, such as iatrogenic fractures or cortical perforations, were not observed. Postoperatively, five patients (2.3%) developed LFCN neuropraxia, and two patients (0.93%) experienced periprosthetic fractures within one year postoperatively. Two cases (0.93%) required revision arthroplasty due to femoral stem subsidence, and one patient (0.47%) was diagnosed with iliopsoas tendinitis. No cases of prosthetic joint dislocations, heterotopic ossifications, or infections were reported (Table 3).

**Table 3:** Comparison of complication rates in the current study and published literature on DAA THA.

Complications	Results in current study *n* (%)	Results from published literature
Transient LFCN neuropraxia	5 (2.3)	0.3–4% [10]
Permanent LFCN neuropraxia	0	0.3–0.8% [11]
Periprosthetic fracture	2 (0.93)	1.2–5.3% [12]
Stem subsidence	2 (0.93)	0.2–0.7% [13]
Iliopsoas tendinitis	1 (0.47)	2.2–5.7% [14]
Dislocation	0	0.3–2.7% [15]
Iatrogenic fracture	0	1.2–5.3% [16]
Leg length discrepancy	0	0.2% [13]
Trochanteric bursitis	0	6.1% [17]
Deep vein thrombosis	0	0.8–1.35% [15]
Canal perforation	0	0.8–9% [18]
Superficial wound infection	0	0.3–4% [19]
Deep wound infection	0	0.3–0.8% [20]

LFCN: lateral femoral cutaneous nerve.

The revision rate for cementless Polar stems was 0.98%, with both revision cases involving these stems. However, Pearson Chi-Square analysis indicated no significant difference in revision rates between Polar and CPCS prosthesis (*p*-value 0.754). Overall revision rate was 0.93%. No revisions were reported for acetabular cup loosening.

### Survival analysis

**Figure 4:**
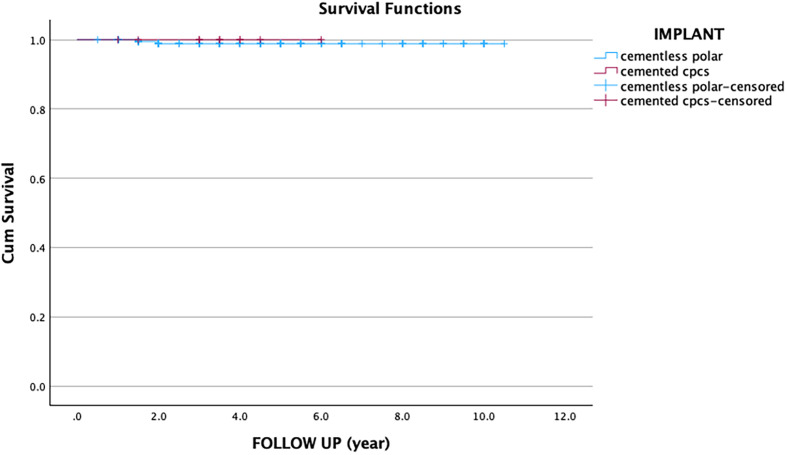
Kaplan-Meier survival curve showing no significant difference in implant survivorship between cementless Polar and cemented CPCS.

Using Kaplan-Meier survival analysis with revision arthroplasty as the defined endpoint at final follow-up, the survival rate of the stem was 99% at an average follow-up of 3.9 years (95% CI: 3.5–4.2 years), while the cup demonstrated a survival rate of 100% at an average follow-up of 3.8 years (95% CI: 3.5–4.2 years) (Figure 4). This data suggests excellent short- to mid-term durability of both the stem and cup components in this cohort.

## Discussion

This study specifically emphasizes the efficacy of the bikini-incision DAA for THA in patients requiring larger acetabular components. Significant improvements in HHS align with existing evidence of DAA’s functional benefits, particularly its association with faster recovery and reduced soft-tissue disruption [21].

The absence of intraoperative complications and low postoperative issues, including neuropraxia (2.3%) and revision arthroplasty (0.93%), reinforce the safety profile of this approach. Additionally, patient factors such as age, BMI, and prosthesis type did not significantly affect outcomes, demonstrating its applicability across diverse demographics and prosthetic designs. However, it is important to discuss potential reasons for the lack of impact of these variables. For example, while BMI and age are often thought to influence surgical outcomes, our findings suggest that the bikini-incision DAA approach can mitigate these effects due to its minimally invasive nature and careful soft tissue handling. Additionally, the absence of significant findings related to these factors might be influenced by sample size or the relatively short-term follow-up period, which limits the assessment of long-term influences.

This study addresses a gap in research on large acetabular cups, mitigating concerns raised in studies reported by Lachiewicz et al. regarding wear or mechanical complications [22]. Our findings indicate similar complications, supporting its safe application. Notably, the low revision rate for cementless Polar stems (0.98%) and absence of acetabular cup loosening underscore the durability of these implants. Furthermore, the exceptional survival rates of the stem (99%) and cup (100%) at a mean follow-up of 3.9 years are consistent with Kaplan-Meier analyses from Prodinger et al. [23]. However, the lack of long-term follow-up data does limit the understanding of potential late complications, such as implant wear or loosening, which could affect the long-term outcomes of this cohort. Longer follow-up periods are needed to fully assess the durability and any delayed complications of the implants.

The complication rates outlined in Table 3 are summarized and compared with published literature on THA.

### Neuropraxia

Transient LFCN neuropraxia was observed in 2.3% of patients in this study, which is within the reported range of 0.3–4% from other studies [10]. This transient condition, while a known complication, typically resolves over time with conservative management. Importantly, no cases of permanent LFCN neuropraxia were observed, which is consistent with reported rates of 0.3–0.8% in the literature [11]. This highlights the careful soft tissue handling in our surgical approach and underscores the potential for the bikini DAA to minimize long-term nerve complications.

All five patients with LFCN neuropraxia reported paraesthesia at the anterolateral thigh, which did not affect daily activities and resolved within six to 18 weeks. To minimize the risk of LFCN injury, the senior author recommends precise surgical techniques. Specifically, the tensor fascia latae (TFL) fascia should be carefully incised lateral to the intermuscular plane during the surgical exposure, ensuring proper positioning before initiating the deep subfascial dissection [4, 8].

### Periprosthetic fractures

The incidence of periprosthetic fractures was 0.93%, which is comparable to the literature, where rates range from 1.2% to 5.3% [12]. This suggests that while periprosthetic fractures are a potential risk, they are relatively rare and can be managed effectively with appropriate fixation techniques.

**Figure 5:**
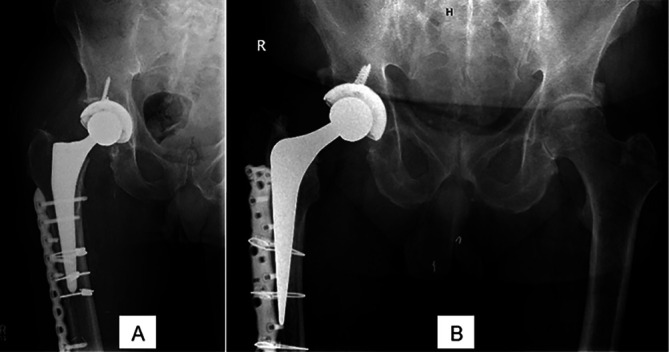
Vancouver B1 Periprosthetic fracture in two patients (A, B), treated with cable plates.

Both cases of periprosthetic fracture were classified as Vancouver B1, allowing the stem’s stability to be preserved. As a result, osteosynthesis was performed using cable plate fixation (Figure 5). This management approach is consistent with existing literature, which suggests that Vancouver B1 fractures typically maintain implant stability and can be treated conservatively with fixation methods such as plating or cable plates, rather than requiring revision surgery [12].

### Femoral stem subsidence

**Figure 6:**
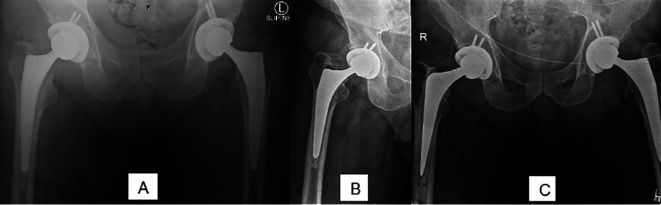
Right femoral stem subsidence in patient with myeloproliferative disorder. (A) Initial plain radiograph. (B) Subsidence at 1.5 year. (C) Plain radiograph post revision arthroplasty with cemented CPCS.

Femoral stem subsidence was also reported in 0.93% of cases, which is slightly higher than the 0.2–0.7% range reported in some studies [13]. However, this remains a low complication rate, and no significant long-term issues were identified in the cohort. Stem subsidence occurred in two patients at 1.5- and two-years post-THA, both involving cementless Polar stems. Revision surgery was performed in both cases, with a cemented CPCS stem used in one patient (Figure 6) and a cemented Echelon revision stem in the other. In the first case, early stem loosening was attributed to an underlying myeloproliferative disorder, which likely compromised bone quality and structural stability, leading to implant failure. In the second case, despite appropriate stem sizing, a low femoral neck cut contributed to stem subsidence over time. The delayed onset of subsidence, occurring only after two years, further supports the role of compromised bone quality in the outcome.

There is a significant link between bone quality, particularly bone mineral density (BMD), and femoral stem subsidence following THA. Studies suggest that low BMD, as seen in conditions such as osteopenia or osteoporosis, compromises the initial stability and osteointegration of cementless femoral stems, making subsidence more likely [24]. In contrast to the patient in the current study, subsidence typically manifests within the first three to six months following surgery [25].

These cases highlight the potential challenges associated with cementless stems in patients with compromised bone health or systemic conditions. Revision with cemented stems provides a viable solution, offering improved fixation and stability in scenarios where bone support is inadequate. This approach aligns with existing literature, which advocates for the use of cemented implants in patients at higher risk of mechanical failure or poor bone integration [25].

One of the challenges frequently encountered with DAA is achieving adequate femoral exposure, which, if insufficient, can lead to complications such as undersized implants, stem subsidence, or early failure of the femoral component [25]. These risks can be mitigated through detailed preoperative templating, systematic soft-tissue release, and careful exposure and handling of the proximal femur. Capsular releases are essential for allowing sufficient external rotation of the femur, facilitating safer access without jeopardizing the abductor muscles. Additionally, elevating the femur with a bone hook improves surgical exposure while reducing the risk of complications [4].

### Iliopsoas tendinitis

Iliopsoas tendinitis occurred in 0.47% of patients, a rate lower than that observed in some studies, which report rates ranging from 2.2% to 5.7% [14]. This relatively low occurrence suggests that the surgical approach and technique may reduce the risk of this complication.

The author highlighted the importance of preserving the anteromedial capsule and avoiding the use of oversized acetabular cups as key strategies in preventing iliopsoas tendinitis, a complication seen in some hip arthroplasty cases [4]. Similar findings have been reported in other studies, which stress the role of precise surgical technique and implant sizing in reducing post-operative complications such as iliopsoas tendinitis [14].

### Intraoperative complications

No iatrogenic fractures or canal perforations were recorded, contrasting with literature-reported rates of 1.2–5.3% for fractures [16] and up to 9% for perforations [18]. These findings emphasize the importance of preoperative planning and careful intraoperative technique in avoiding these complications.

### Dislocation, infections, and other complications

No cases of dislocation, infection, or significant leg length discrepancy were reported, which aligns with low rates of these complications seen in other studies (dislocations: 0.3–2.7%, infections: 0.3–4%) [15, 19, 20]. The absence of these complications supports the safety and reliability of the surgical technique and implant selection.

## Study limitations

While this study provides valuable insights into the safety and outcomes of the bikini-incision DAA for THA, several limitations must be considered. Firstly, the absence of a randomized controlled design and the lack of a control group introduce potential selection bias, which may impact the reliability and generalizability of the findings. As all procedures were performed by a single surgeon, variability in surgical technique and experience could also affect the outcomes. This single-surgeon series may not reflect the broader outcomes seen in multi-surgeon practices, where variations in technique and proficiency are more pronounced. The potential for confounding factors that were not controlled for, such as differences in surgeon experience, intraoperative management, or the absence of a control cohort, could also introduce bias in the results.

Additionally, the study’s retrospective design limits the ability to draw definitive conclusions regarding causality. The relatively small sample size, due to the study’s focus on patients requiring large acetabular cups, also reduces the statistical power and generalizability of the results. While the findings provide useful information, they may not fully represent the outcomes in the wider patient population, particularly those with smaller cups or other prosthetic designs.

This study primarily focused on short- to mid-term outcomes, leaving long-term data on wear patterns, implant longevity, and potential complications unaddressed. This gap in long-term follow-up data restricts the ability to assess late complications such as implant loosening, wear, or osteolysis, which are critical to understand the full durability of the implants. Longer-term studies are needed to evaluate the true longevity of the prostheses.

Furthermore, the lack of subgroup analysis based on patient-specific factors, such as body mass index (BMI), bone quality, or activity level, restricts the ability to draw conclusions about the broader applicability of the findings across diverse patient populations. Future studies should consider stratifying patients based on these variables to better assess the impact of individual factors on outcomes. Additionally, a more comprehensive assessment of potential confounding factors, including pre-existing conditions or comorbidities, would provide a clearer understanding of how these factors influence surgical results and recovery.

Finally, one significant limitation not fully addressed in this study is the potential learning curve for surgeons adopting the bikini-incision DAA approach. The technique is still being refined, and less experienced surgeons may experience a higher incidence of complications or longer operation times compared to those with more experience. The results presented in this study may reflect the outcomes of a surgeon with significant experience in DAA, which may not be representative of early-stage outcomes for less-experienced surgeons. Future research should incorporate multi-centre studies with a wider range of surgeon experience levels to better understand how the learning curve impacts outcomes.

While this study offers valuable insights, future research with a larger, multi-centre, randomized cohort and longer follow-up will be necessary to fully understand the long-term effects and to mitigate the potential biases and confounding factors present in this analysis.

## Conclusion

This study demonstrates that the bikini incision DAA for THA using large acetabular cups (≥56 mm) is a safe and effective procedure. Significant improvements in HHS reflect excellent functional outcomes, while low complication and revision rates highlight its safety profile. The survival rates of the prosthetic components were exceptional, with no cases of cup loosening. These findings support the use of this approach in patients requiring larger prostheses. However, the lack of a control group and the retrospective design limit the ability to draw definitive conclusions. Therefore, prospective randomized studies with control groups and longer follow-up periods are essential to validate these results and assess the long-term safety, durability, and functional outcomes of this technique.

## Data Availability

Data and materials are available on request.
